# Retraction Note: Comparison of traditional and new generation DNA markers declares high genetic diversity and differentiated population structure of wild almond species

**DOI:** 10.1038/s41598-020-72522-5

**Published:** 2020-09-22

**Authors:** Karim Sorkheh, Mehrana Koohi Dehkordi, Sezai Ercisli, Attila Hegedus, Júlia Halász

**Affiliations:** 1 Department of Agronomy and Plant Breeding, Faculty of Agriculture, Shahid Chamran University of Ahvaz, P.O. Box 61355/144, Ahvaz, Iran; 2 Department of Agronomy, Faculty of Agriculture, Payame-Noor University, P.O. Box 19395-3697, Tehran, Iran; 3 Department of Horticulture, Agricultural Faculty, Ataturk University, 25240 Erzurum, Turkey; 4 Department of Genetics and Plant Breeding, Szent István University, Villányiút 29-43, 1118 Budapest, Hungary

Retraction of: *Scientific Reports* 10.1038/s41598-017-06084-4, published online 20 July 2017

The Editors have retracted this article.

After publication concerns were raised that there were anomalies in Supplementary Figures 1 and 2. However, the authors were unable to provide higher resolution images to allow the veracity of these data to be confirmed. Additionally the graph showing the genetic diversity of the wild almond species, which is a part of Figures 1, 2, 3 and 5, appears to be a duplication of a graph showing the genetic diversity of the liquorice species in Figure 2 in Hakimi et al.^1^, of the barley species in Figure 2 in Rouhain et al.^2^, and of olive species in Figures 1 and 2 in Khaleghi et al.^3^.

The Editors therefore no longer have confidence in the validity of the results and conclusions reported in this article.

All authors agree with the retraction.
